# Hepatic dysfunction and thrombocytopenia induced by excess sFlt1 in mice lacking endothelial nitric oxide synthase

**DOI:** 10.1038/s41598-017-18260-7

**Published:** 2018-01-08

**Authors:** Yuji Oe, Mieko Ko, Tomofumi Fushima, Emiko Sato, S. Ananth Karumanchi, Hiroshi Sato, Junichi Sugawara, Sadayoshi Ito, Nobuyuki Takahashi

**Affiliations:** 1 Division of Feto-Maternal Medical Science, Department of Community Medical Support, Tohoku Medical Megabank Organization, Tohoku University, Sendai, 980-8574 Japan; 2 Division of Clinical Pharmacology and Therapeutics, Tohoku University Graduate School of Pharmaceutical Sciences & Faculty of Pharmaceutical Sciences, Sendai, 980-8578 Japan; 3 Division of Nephrology, Endocrinology, and Vascular Medicine, Tohoku University Graduate School of Medicine, Sendai, 980-8574 Japan; 4 Department of Medicine and Center for Vascular Biology Research, Beth Israel Deaconess Medical Center and Harvard Medical School, Boston, MA 02215 USA; 5 Research Fellow of Japan Society for the Promotion of Science, Chiyoda-ku, Tokyo 102-0083 Japan

## Abstract

Liver dysfunction is a major problem in patients with severe preeclampsia (PE), hemolysis, elevated liver enzymes, and low platelet count (HELLP) syndrome, or in patients receiving anti-vascular endothelial growth factor (VEGF) therapy. Excessive soluble fms-like tyrosine kinase 1 (sFlt1) that antagonizes VEGF has been implicated in the pathogenesis of PE. VEGF increases the expression of endothelial nitric oxide synthase (eNOS) and activates it. *eNOS* polymorphisms that cause reduced NO production are associated with PE. The aim of this study was to clarify the role on hepatic function by excess sFlt1 in the absence of *eNOS* gene product. We first overexpressed sFlt1 using adenovirus in *eNOS* ^−/−^ and *eNOS* ^+/+^ mice. Excessive sFlt1 and lack of *eNOS* synergistically increased plasma levels of liver transaminases, exacerbated infiltration of inflammatory cells, elevated expression levels of cytokines in the liver, and aggravated oxidative stress and coagulation abnormalities. Lack of *eNOS* in the presence of excess sFlt1 also induced thrombocytopenia, whereas *eNOS* ^+/+^ mice with excess sFlt1 alone showed no or modest liver phenotype. Taken together, excessive sFlt1 and lack of *eNOS* synergistically induce hepatic dysfunction and thrombocytopenia, suggesting a novel role for VEGF and nitric oxide signaling in hepatocyte-endothelial cross-talk in health and in liver injury states.

## Introduction

Vascular endothelial growth factor (VEGF) is indispensable in the maturation and maintenance of endothelial cells^1^. VEGF produced by hepatocytes acts on VEGF receptors expressed in sinusoidal endothelial cells, and is essential for maintaining liver homeostasis^2,3^. Although inhibitors of VEGF signaling are widely used as anti-cancer therapy, their hepatotoxicity is problematic^4^. Moreover, placental upregulation of endogenous sFlt1 that acts as an inhibitor of VEGF and placental growth factor (PlGF) signaling, has been linked to the pathogenesis of preeclampsia (PE)^5,6^ and possibly hemolysis, elevated liver enzyme levels, and low platelet levels (HELLP) syndrome that exhibits liver dysfunction^7,8^.

The effect of inhibiting VEGF on liver injury has not been well studied. Some investigators showed that knocking down hepatic VEGF or excessive sFlt1 causes hepatotoxicity^2,9^, whereas others have demonstrated VEGF inhibitors do not affect liver function^10^. These findings may indicate that additional factor(s) may be required for VEGF inhibitors to induce liver dysfunction.

VEGF activates eNOS through phosphorylation of Ser 1177^11^. Typical *eNOS* gene polymorphisms, G894T and T-786C, are associated with the onset of PE or HELLP syndrome^12,13^. Consistent with these findings, it was recently reported that hypertension and placental ischemia induced by sFlt1 may be dependent on impaired NO signaling, and that sildenafil, a cyclic GMP agonist, can reverse sFlt1 mediated adverse pregnancy outcomes^14^. However, it is likely that sFlt1 also induces several NO independent pathways. In this regard, we have previously demonstrated that lack of *eNOS* exacerbates sFlt1-induced kidney injury through endothelin activation^15^. Based on these findings, we hypothesized that eNOS dysfunction is likely involved in the exacerbation of tissue injury caused by VEGF inhibition.

Here, we demonstrate that excessive sFlt1 combined with lack of *eNOS* in non-pregnant mice causes severe liver dysfunction accompanied by hepatic inflammation, oxidative stress, and dyslipidemia. Coagulation abnormalities and thrombocytopenia were also evident.

## Results

### Characteristics and liver dysfunction induced by excessive sFlt1 in mice lacking eNOS

**Figure 1:**
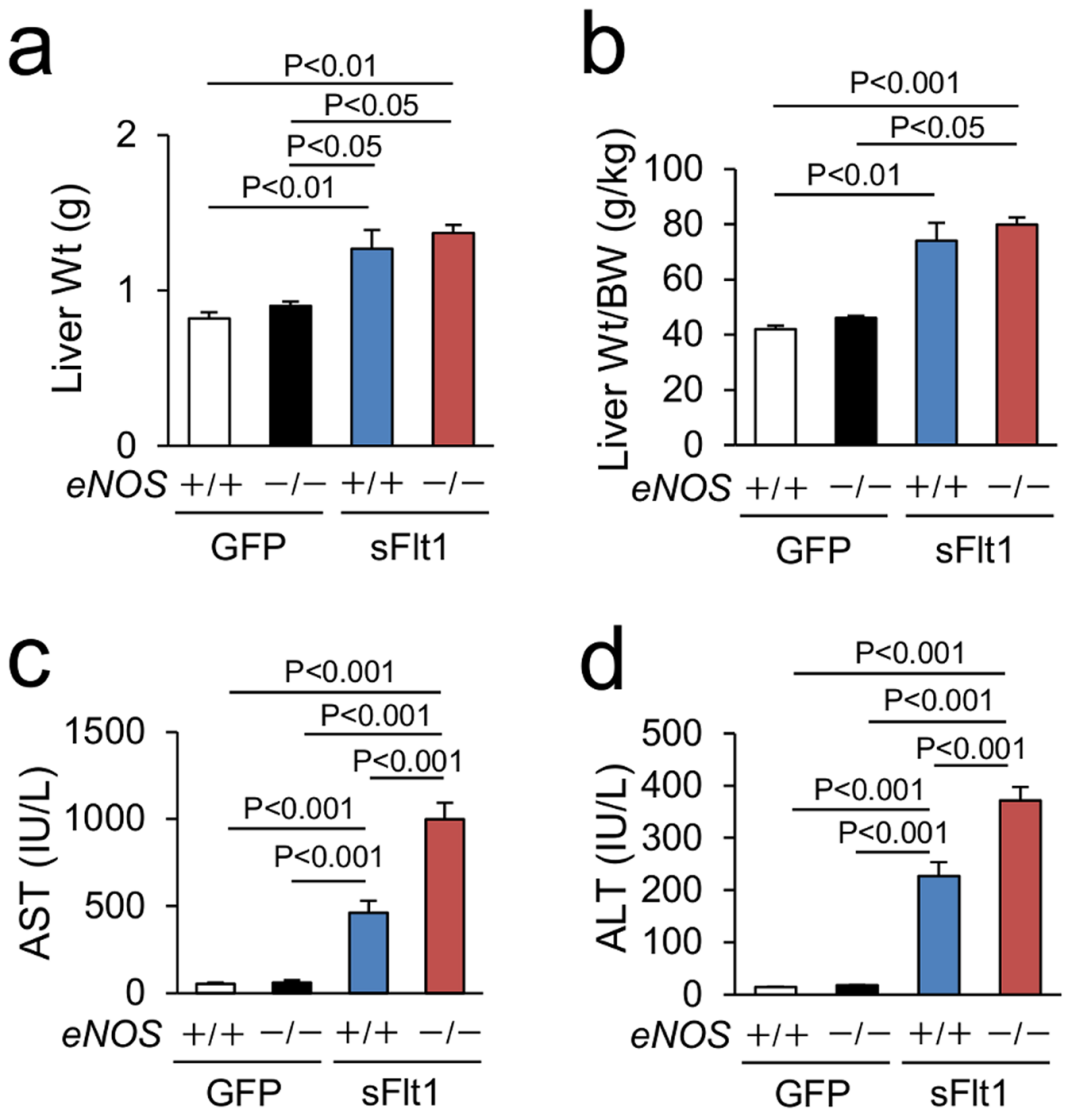
Liver dysfunction induced by excessive sFlt1 in mice lacking *eNOS*. (**a**) Liver weight (Wt). (**b**) Liver Wt/body weight (BW). Excessive sFlt1 causes hepatomegaly. The levels of plasma Aspartate transaminase (AST) and Alanine transaminase (ALT) are severely increased in *eNOS* ^−/−^; sFlt1 mice (**c**,**d**) n = 7–9. Data are shown as mean ± s.e.m. ANOVA or Kruskal-Wallis test.

We used non-pregnant *eNOS* ^*−/−*^ mice overexpressing sFlt1. *eNOS* ^−/−^; sFlt1 mice showed severe glomerular injury and massive albuminuria that is consistent with our previous observation (Supplementary Figure 1a,b)^15^. Liver weight and liver weight/body weight were larger in mice with excessive sFlt1 (Fig. 1a,b). Lack of *eNOS* did not affect them. In contrast, levels of plasma aspartate transaminase (AST) were significantly elevated in *eNOS* ^−/−^; sFlt1 (998.5 ± 96.8 IU/L) compared to those of controls (55.1 ± 6.3 IU/L in *eNOS* ^+/+^ and 63.8 ± 11.5 IU/L in *eNOS* ^−/−^) and of *eNOS* ^+/+^; sFlt1 mice (463.0 ± 68.5 IU/L) (Fig. 1c). The levels of alanine transaminase (ALT) were also elevated in *eNOS* ^−/−^; sFlt1 (371.1 ± 26.4 IU/l) compared to those of controls (14.7 ± 0.6 IU/L in *eNOS* ^+/+^ and 17.8 ± 0.5 IU/L in *eNOS* ^−/−^) and *eNOS* ^+/+^; sFlt1 mice (227.2 ± 26.4 IU/L) (Fig. 1d). These data were specific to sFlt1 overexpression as GFP overexpressing control mice did not demonstrate any hepatic phenotype (Fig. 1a–d).

### Histological damage and inflammation in the liver

**Figure 2:**
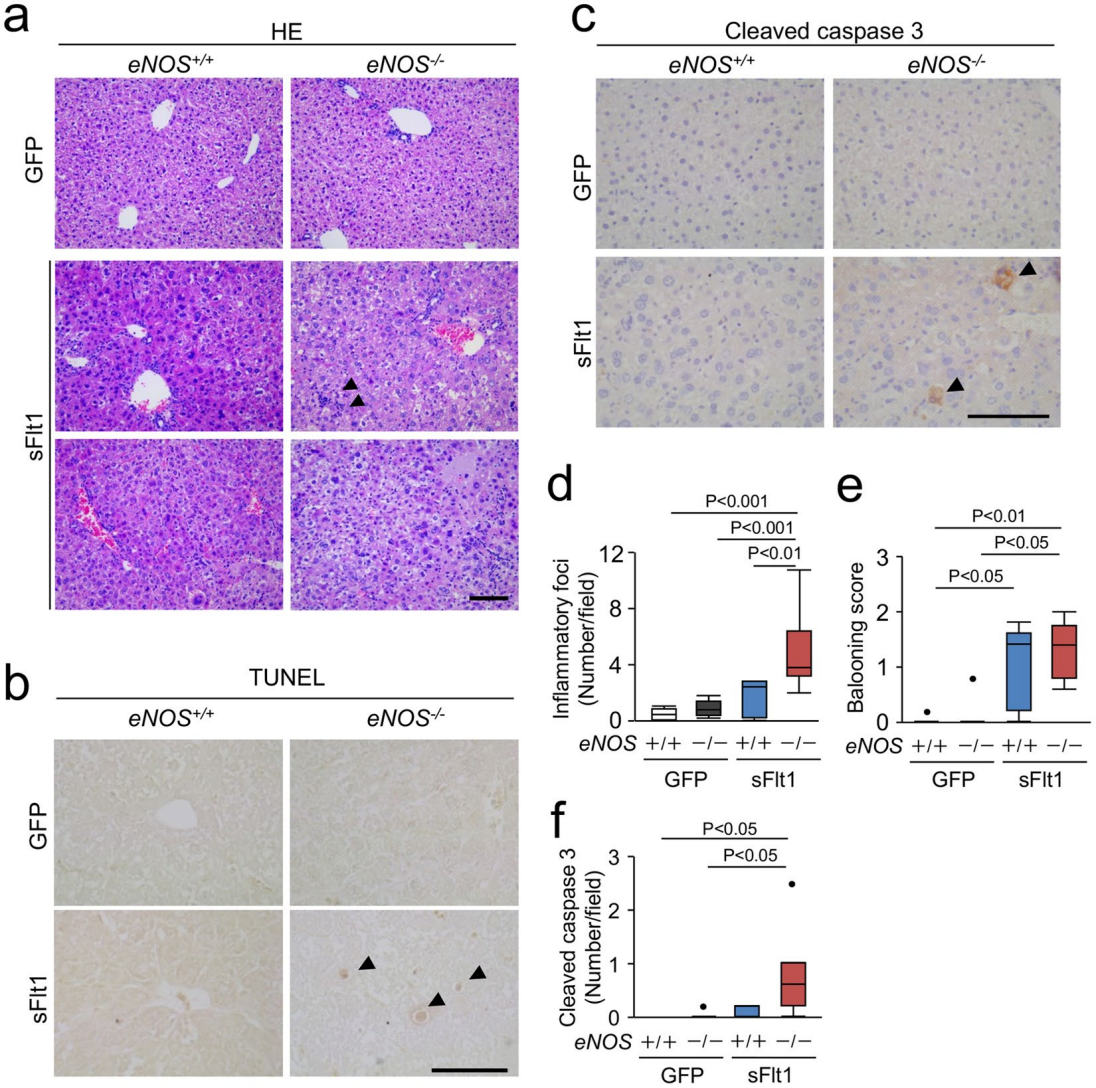
Histological damage in the liver. (**a**) Representative photomicrographs of Hematoxylin Eosin (HE). Inflammatory foci (arrowheads), vacuolar degeneration, and necrosis are shown in the liver from *eNOS* ^−/−^; sFlt1 mice. TUNEL (**b**) and immunohistochemistry against cleaved caspase 3 (**c**) in the liver. Scale bar indicates 100 µm. (**d**) The number of inflammatory foci is significantly increased in the liver from *eNOS* ^−/−^; sFlt1 mice. (**e**) The score of ballooning hepatocytes. (**f**) Increased cleaved caspase 3 positive cells in the liver from *eNOS* ^−/−^; sFlt1 mice. n = 5–8. Data are shown as box plot. ANOVA or Kruskal-Wallis test.

Since lack of *eNOS* in mice with excessive sFlt1 further increased the levels of ALT and AST, we next performed pathological analysis of the liver. Figure 2a–c shows the representative hepatic photomicrographs of Hematoxylin-Eosin stain, TdT-mediated dUTP nick end labeling (TUNEL) stain and immunohistochemistry against cleaved caspase 3. The liver from *eNOS* ^−/−^; sFlt1 mice exhibited hepatocyte ballooning accompanied by vacuolar degeneration, necrotic lesion, and infiltration of inflammatory cells (Fig. 2a). The number of inflammatory foci was significantly increased in *eNOS* ^−/−^; sFlt1 mice compared to that of control and *eNOS* ^+/+^; sFlt1 mice (Fig. 2d). There was an increase in the hepatocyte ballooning score with excessive sFlt1 in both *eNOS* ^+/+^ mice and *eNOS* ^−/−^ mice (Fig. 2e). The cleaved caspase 3 positive hepatocytes were frequently observed in the liver from *eNOS* ^−/−^; sFlt1 mice (Fig. 2f).

**Figure 3:**
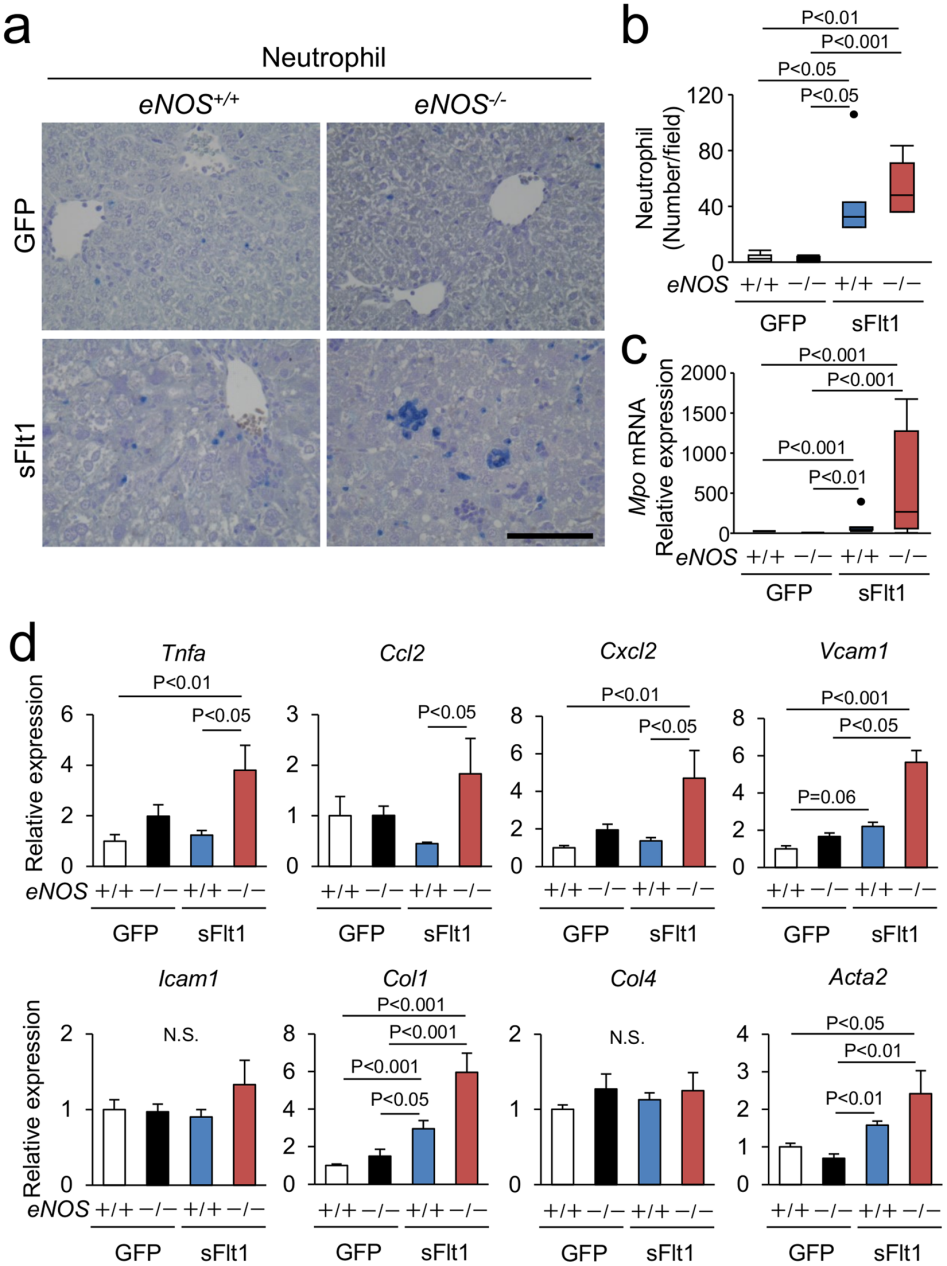
Inflammation in the liver. (**a**,**b**) Infiltrating neutrophils are visualized by Naphthol AS-D chloroacetate Esterase stain (blue). Scale bar indicates 100 µm. Number of neutrophil is increased by excessive sFlt1, which is further up-regulated by *eNOS* deletion. (**c**) The level of *Mpo* (myeloperoxidase) mRNA drastically increased in the liver from *eNOS* ^−/−^; sFlt1 mice. (**d**) Expression of inflammation and pro-fibrotic related genes in the liver. N.S., not significant. n = 7–8. Data are shown as mean ± s.e.m or box plot. ANOVA or Kruskal-Wallis test.

To further analyze the inflammation in the liver, we tested the changes in neutrophil infiltration and the expression of proinflammatory and profibrotic genes in the liver. Excessive sFlt1 in the *eNOS* ^−/−^ mice increased the number of infiltrating neutrophils (Fig. 3a,b). The levels of hepatic myeloperoxidase (*Mpo*) mRNA in the *eNOS* ^−/−^; sFlt1 mice were more than 500 fold higher than that of the *eNOS* ^+/+^ mice (Fig. 3c), whereas excessive sFlt1 per se did not affect macrophage infiltration (Supplementary Figure 2a,b). As shown in Fig. 3d, lack of *eNOS* elevated the expression levels of *Tnfa, Ccl2, Cxcl2* and *Vcam1* only in the setting of excessive sFlt1. Similarly, the levels of pro-fibrotic genes, *Col1* and *Acta2*, were elevated in the *eNOS* ^−/−^; sFlt1 mice (Fig. 3d). These findings indicate that in the setting of excessive sFlt1, lack of *eNOS* exacerbates histological damage and inflammation in the liver.

### Oxidative stress and hypoxia

**Figure 4:**
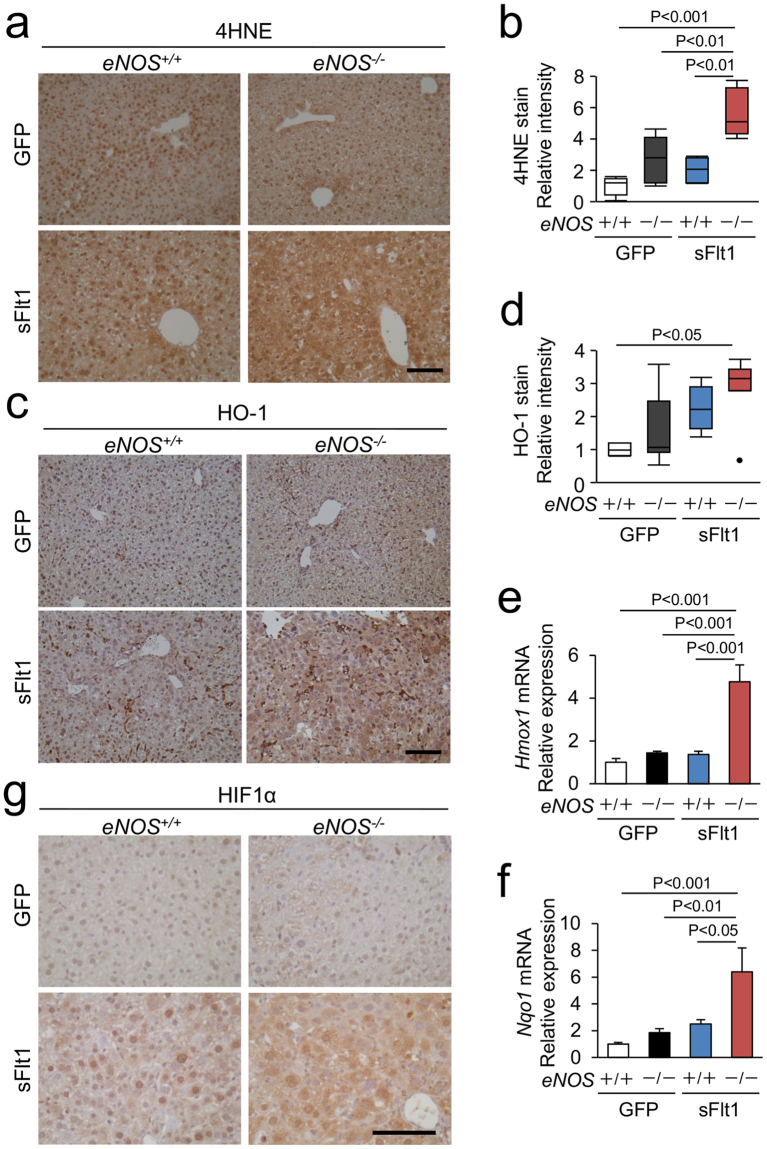
Markers of oxidative stress and hypoxia in the liver. (**a**) Representative photomicrographs of immunohistochemistry against 4-hydroxy-2-nonenal (4HNE). (**b**) Strong immunoreactive 4HNE is shown in the liver from *eNOS* ^−/−^; sFlt1 mice. (**c**) Representative photomicrographs of immunohistochemistry against HO-1. (**d**) Strong immunoreactive HO-1 is shown in the liver from *eNOS* ^−/−^; sFlt1 mice. (**e**,**f**) The levels of *Hmox1* and *Nqo1* mRNA in the liver. (**g**) Representative photomicrographs of immunohistochemistry against Hypoxia inducible factor 1α (HIF1α) in the liver. n = 5–8. Data are shown as mean ± s.e.m or box plot. ANOVA or Kruskal-Wallis test.

Inhibition of VEGF or of eNOS exacerbates hepatic hypoxia pathways^2,16^, which increases oxidative stress and promotes liver injury^17^. Accordingly, we examined oxidative stress and hypoxia in this model. Intensity of immunoreactive 4-hydroxy-2-nonenal (4HNE) in the liver from the *eNOS* ^−/−^; sFlt1 mice was significantly higher than that from the *eNOS* ^+/+^ mice with or without excessive sFlt1, suggesting that oxidative stress is increased in the liver from the *eNOS* ^−/−^; sFlt1 mice (Fig. 4a,b). The protein and gene expression of HO-1 (*Hmox1*), an anti-oxidative enzyme, was increased in the liver from the *eNOS* ^−/−^; sFlt1 mice (Fig. 4c–e). Similarly, gene expression of *Nqo1* was up-regulated (Fig. 4f). Strong immunoreactive hypoxia inducible factor 1α (HIF1α) was observed in the liver from the *eNOS* ^−/−^; sFlt1 mice (Fig. 4g). Moreover, the gene expression of *Glut1* and *Epo*, other typical target genes of HIF, was significantly upregulated in the *eNOS* ^−/−^; sFlt1 mice (Supplementary Figure 3a,b). We conclude that lack of *eNOS* in mice with excessive sFlt1 aggravates oxidative stress and hypoxia, which likely induces severe liver injury.

### Lipid metabolism in the liver

**Figure 5:**
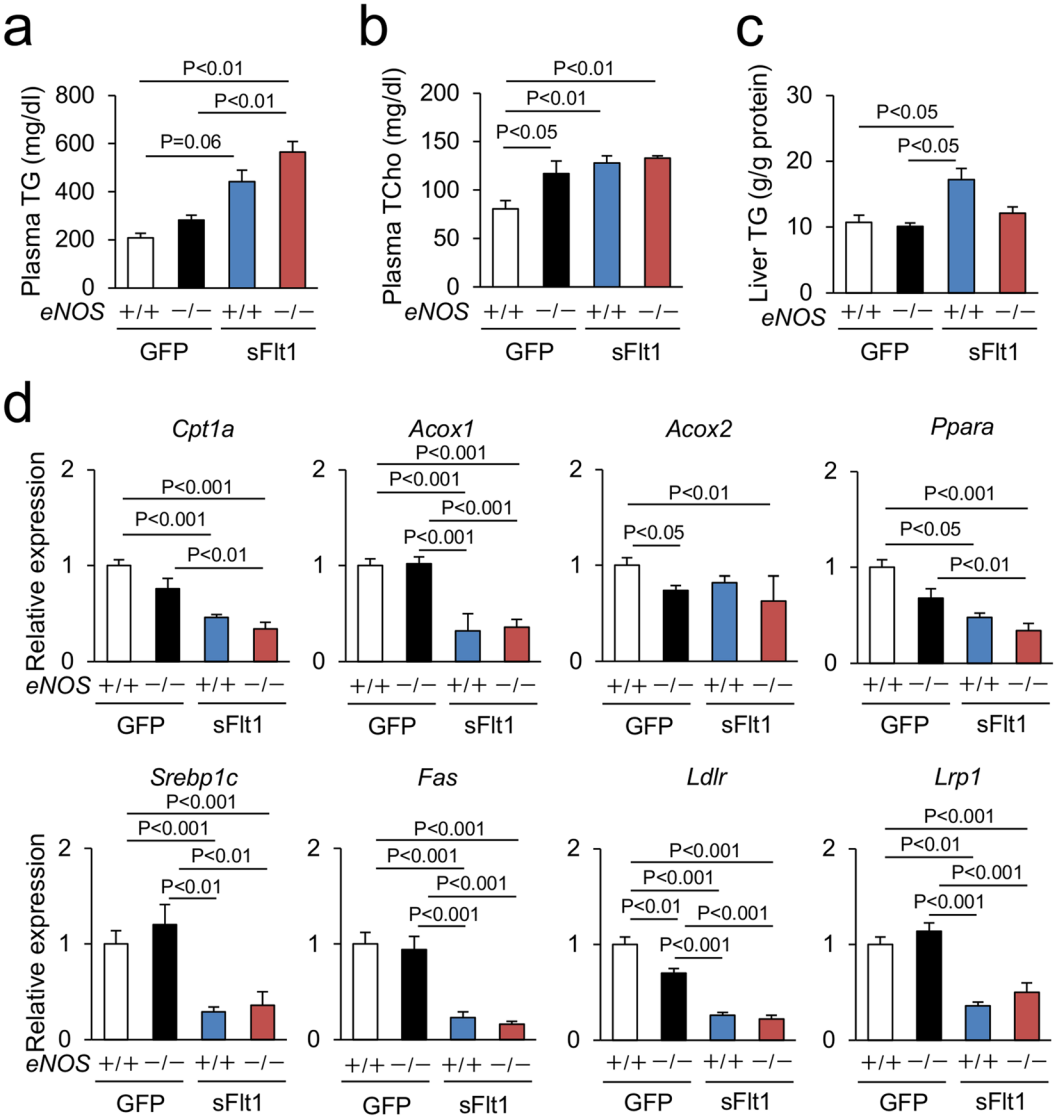
Lipid metabolism in the liver. (**a**–**c**) Plasma triglyceride (TG), total cholesterol (TCho), and hepatic TG. (**d**) Expression of Fatty acid oxidation, lipogenesis, and lipoprotein clearance receptor related genes in the liver, which are down-regulated by excessive sFlt1. n = 7–8. Data are shown as mean ± s.e.m. ANOVA or Kruskal-Wallis test.

Liver plays a central role in lipid metabolism, and dyslipidemia is commonly observed in preeclamptic women^18,19^. Accordingly, we quantified lipid parameters in the four groups of mice. Excessive sFlt1 increased the levels of plasma triglyceride, plasma total cholesterol, and liver triglyceride content in the wild type *eNOS* mice, but lack of *eNOS* did not affect these parameters in mice with excessive sFlt1 (Fig. 5a–c). The expression levels of key regulators of hepatic lipid metabolism, fatty acid oxidation (*Cpt1a*, *Acox1*, and *Ppara*), lipogenesis (*Srebp1c* and *Fas*), and lipoprotein clearance receptors (*Ldlr* and *Lrp1*) were reduced by excessive sFlt1, but lack of *eNOS* did not affect them (Fig. 5d).

### Thrombocytopenia induced by excessive sFlt1 in mice lacking eNOS

**Figure 6:**
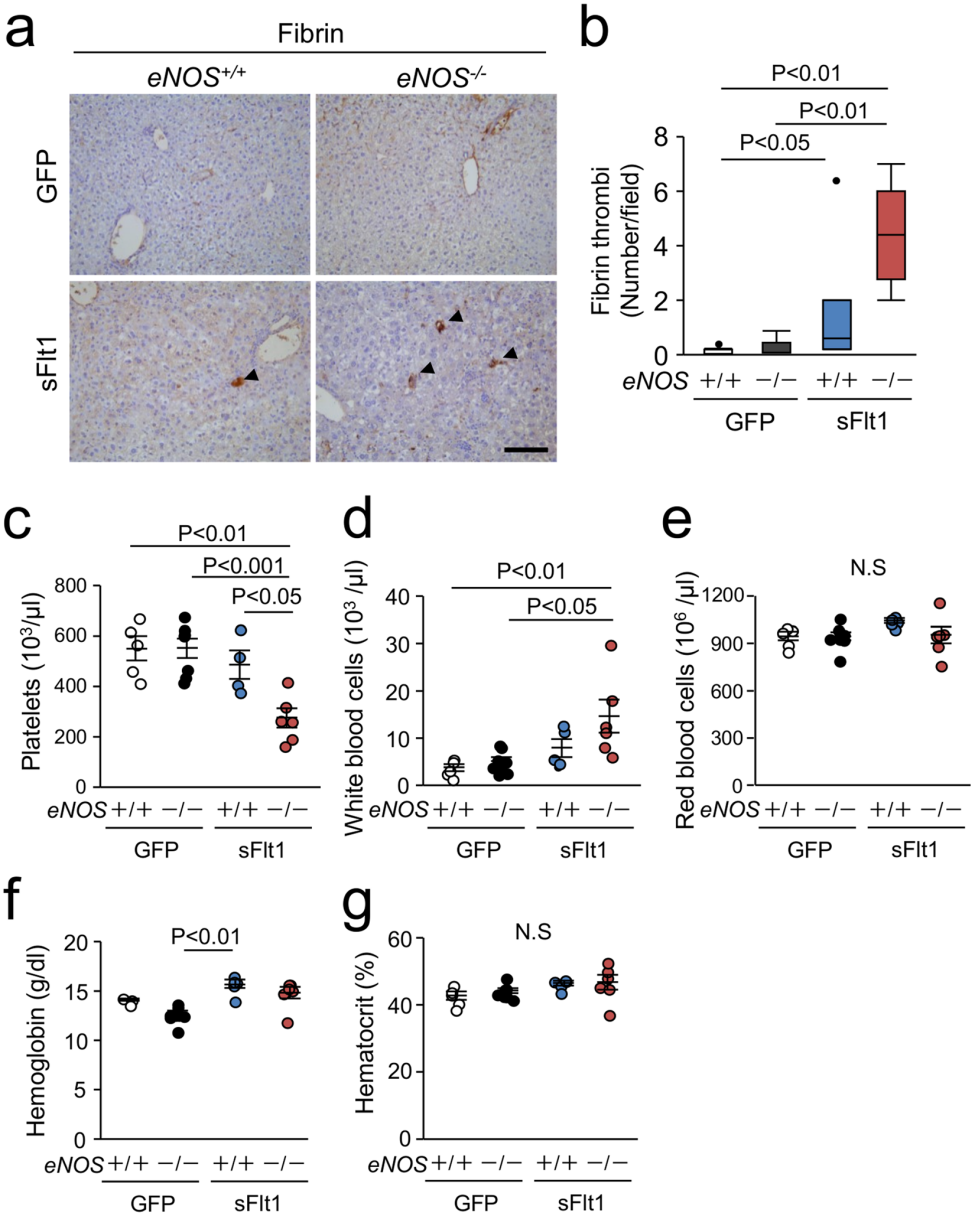
Fibrin deposition in the liver and thrombocytopenia. (**a**) Representative photomicrographs of immunohistochemistry against Fibrin. Scale bar indicates 100 µm. (**b**) Number of Fibrin thrombi is significantly increased in the liver from *eNOS* ^−/−^; sFlt1 mice. (**c**–**g**) Data of blood count; platelets (**c**), white blood cells (**d**), red blood cells (**e**), hemoglobin (**f**), and hematocrit (**g**). Excessive sFlt1 combined *eNOS* deletion causes thrombocytopenia. N.S., not significant. n = 4–8. Data are shown as mean ± s.e.m or box plot. ANOVA or Kruskal-Wallis test.

Because VEGF inhibitor therapies and severe preeclampsia is characterized by hematological abnormalities^20,21^, we next examined hematological parameters in this model. The number of fibrin thrombi was significantly higher in the liver from the *eNOS* ^−/−^; sFlt1 mice than that from other three groups of mice (Fig. 6a,b). Complete blood count showed reduced platelet number and increased white blood cell number in the *eNOS* ^−/−^; sFlt1 mice (Fig. 6c,d). The red blood cell count and hematocrit were similar among the groups (Fig. 6e–g). These findings indicate that lack of *eNOS* in mice with excessive sFlt1 caused hypercoagulability and thrombocytopenia. Although inhibiting VEGF causes thrombotic microangiopathy and hemolytic anemia^22,23^, excessive sFlt1 and lack of *eNOS* did not cause anemia. Consistent with this observation, the level of plasma haptoglobin, a marker of hemolysis, was not statistically different between the groups (Supplementary Figure 4). Moreover, schistocytes were not observed in their smears (data not shown). We conclude that lack of *eNOS* in the context of excessive sFlt1 exacerbates hypercoagulability and thrombocytopenia without obvious hemolysis.

## Discussion

We have demonstrated that the lack of *eNOS* in the presence of excessive sFlt1 exacerbates hepatic injury and causes hypercoagulability and thrombocytopenia. Our data show that the livers from the *eNOS* ^−/−^; sFlt1 mice have enhanced hepatic inflammation, prominent neutrophil infiltration, and increased oxidative stress and the expression of genes induced by hypoxia. Literature shows that nitric oxide (NO) derived from eNOS is anti-inflammatory *in vitro* and *in vivo* ^16,24–26^. NO-donor directly reduces the expression levels of hypoxia-induced cytokines and chemokines in HepG2 cells^24^. Lack or inhibition of eNOS exacerbates hepatic inflammation in obesity and ischemic models^16,25,26^. Moreover, hepatocyte specific deletion of VEGF causes hypoxia^2,27^, which causes tissue injury mediated by HIF^28^. Up-regulated HIF1α and HIF2α increase hepatic inflammation, and contribute to alcoholic or non-alcoholic liver disease and acetaminophen induced liver injury^29–31^. Consistent with these findings, our data suggest that inflammation, hypoxia, and oxidative stress could be an important pathogenic factor in the exacerbation of liver injury in setting of reduced VEGF signaling and impaired NO production.

In our experimental condition, adenovirus increased plasma sFlt1 concentration to ~1.0 × 10^4^ ng/ml (Supplementary Figure 5). Previous report demonstrated that such a high level of sFlt1 almost completely inhibits VEGF signaling^10^. Furthermore, the lack of any phenotype in the control adenoviral group suggests that the phenotype induced by sFlt1 is specific to VEGF inhibition. Moreover, eNOS dysfunction is likely crucial to the onset or exacerbation of VEGF inhibitor-induced liver injury, because wild type *eNOS* mice with extremely excessive sFlt1 did not show hepatic damage.

The patients with PE and HELLP syndrome have elevated levels of serum triglyceride and fatty acid compared to those of normal pregnancy^18,19,32^, suggesting that inhibiting VEGF is associated with abnormal lipid metabolism in the liver. In accordance with this finding, our data indicate that sFlt1 overexpression increases the levels of plasma triglyceride and total cholesterol. However, lack of *eNOS* did not further exacerbate these parameters (Fig. 5). Literature shows that hepatocyte specific inhibition of triglyceride-rich lipoprotein clearance receptors *Ldlr* or *Lrp*, elevated plasma lipoprotein^33^, and that skeletal muscle and adipose tissue actively regulates lipoprotein clearance^34^. Consistent with these findings, excessive sFlt1 reduced the expression levels of *Ldlr* and *Lrp1* in the liver (Fig. 5d). Despite liver damage and abnormal lipid profile in the plasma, the liver did not show increased triglyceride content in the *eNOS* ^−/−^; sFlt1 mice. It is likely that reduced fatty acid oxidation in the liver suppresses lipogenesis and lipid uptake in the liver, leading to increased plasma triglyceride levels. There was no remarkable effect of lack of *eNOS* on lipid metabolism in the liver.

Thrombocytopenia is a characteristic feature of VEGF inhibitor-induced thrombotic microangiopathy^22^, but its pathogenesis remains unclear. Previous report demonstrates that excessive sFlt1 together with lack of *Adamts13* develops hemolysis and thrombocytopenia in mice^35^. Pregnant mice with excessive sFlt1 and soluble endoglin mimic features of human HELLP syndrome^8^. Our data and these findings suggest that excessive sFlt1 alone is not sufficient to cause thrombocytopenia. Because NO derived from eNOS inhibits platelet activation^24,36^, we suggest that reduced NO from eNOS causes thrombocytopenia when VEGF is inhibited.

Stringent VEGF inhibition increases hepatocyte erythropoiesis and polycythemia, which is mediated by increased erythropoietin production due to HIF2 activation^10,37^. But this was not evident in our model, although the levels of *Epo* mRNA in the liver were elevated with excessive sFlt1 (Supplementary Figure 4b). Overexpression of both sFlt1 and soluble endoglin displays the phenotype of HELLP syndrome including hemolysis^8^. Excessive sFlt1 together with lack of *eNOS* is not sufficient to cause hemolysis, and overexpression of both sFlt1 and soluble endoglin is likely necessary for hemolysis to cause HELLP syndrome. However, some preeclamptic patients have liver injury and thrombocytopenia without hemolysis^38^, and our model could explain the pathogenesis of these patients.

sFlt1 is known to inhibit PlGF signaling. However, PlGF is largely made during pregnancy and at least 7–8 folds lower in non-pregnant states^39^. Moreover, lack of *Plgf* does not affect normal angiogenesis, and PlGF blockade rather ameliorates liver fibrosis and inflammation in cirrhotic mice^40–42^. Hepatic expression of PlGF is undetectably low in our preliminary observation and in prior reports^40,42^. Accordingly, we believe inhibition of PlGF does not contribute to exacerbation of liver toxicity by excessive sFlt1 in our model. However, whether this is true during pregnancy where PlGF is abundantly made needs additional studies.

We used non-pregnant mice with excessive sFlt1 because increased sFlt-1 recapitulates the phenotype of maternal syndrome of preeclampsia regardless of pregnancy in rodent models^6,15^. However, sFlt1 explains only some aspects of the pathogenesis of preeclampsia. Various factors including endoglin, endothelin, catechol-O-methyltransferase, or angiotensin-II are likely involved in endothelial dysfunction in pregnant or preeclampsia condition^8,15,43,44^. Their interaction with eNOS and the role in hepatic injury should be clarified in the future.

In conclusion, we have demonstrated that hepatotoxicity of sFlt1 is exacerbated by lack of *eNOS*. Further studies should evaluate the nitric oxide independent pathways induced by VEGF inhibition. These findings might open a novel role of hepatocyte-endothelial communication in the liver homeostasis and underling mechanism of liver injury induced by impaired VEGF signaling.

## Methods

### Animals

All experiments were conducted in compliance with the guidelines of Tohoku University. Experimental protocol was approved by the Institutional Animal Care and Use Committee at Tohoku University. Ten to fourteen-week-old non-pregnant female *eNOS* ^*−/−*^ mice with C57BL/6 J genetic background were injected with 1 × 10^9^ PFU adenovirus to overexpress sFlt1 (Adeno sFlt1) or adenovirus encoding GFP protein (Adeno GFP) at equivalent doses as we previously described^15,45^. These mice were maintained for 7 days. Previous studies have shown that increased sFlt-1 recapitulates the phenotype of preeclampsia regardless of whether the animal is pregnant^6,15,45^. Mice cannot maintain pregnancy if excessive sFlt-1 and lack of *eNOS* are combined (our unpublished observation)^15^. Accordingly, we used non-pregnant female *eNOS* ^*−/−*^ mice for these studies.

### Biochemical measurement

ELISA kits were used to measure urinary albumin (Exocell Inc., Philadelphia, PA), plasma sFlt1 (R&D Systems Inc, Minneapolis, MN) and plasma haptoglobin (Life Diagnostics, Inc. West Chester, PA). Colorimetric detection kits were used to measure AST, ALT, triglyceride and total cholesterol (Wako chemicals, Osaka, Japan) in plasma and liver homogenate. Urinary creatinine was determined by the method we developed using LC-MS/MS^46^.

### Blood count

Blood was collected with EDTA and analyzed using Microsemi LC-662 (Horiba, Japan).

### Quantitative RT-PCR

Total RNA from the liver was extracted using TRI Reagent (Molecular Research Center, Inc., Cincinnati, OH). Hypoxanthine-guanine phosphoribosyltransferase (*Hprt*) was used as a reference gene as we previously reported^47,48^. The primers used in this study have been previously described elsewhere. Their sequences are available on request.

### Morphological study

Livers were fixed in 2% PFA and embedded in paraffin. The sections 2 μm in thickness were stained with Hematoxylin-Eosin stain to evaluate histological damage. The degree of lobular inflammation was evaluated by counting of inflammatory foci. The degree of Injured hepatocytes was examined using ballooning score as previously described^49^. Ballooning score was determined according to number of ballooned hepatocytes: 0 (none), 1 (few), and 2 (many). 5 consecutive fields were examined in each slide at 100-fold magnification. All examination was performed under blinded manner.

### Immunohistochemistry

For immunohistochemistry, rabbit anti-human cleaved caspase 3 antibody (1:300, Cell Signaling Technology, Danvers, MA), rabbit anti-human hypoxia-inducible factor 1a antibody (1:1000, Novus Biologicals, Littleton, CO), anti-human 4-hydroxy-2-nonenal antibody (10 µg/ml, Japan Institute for the Control of Aging, Japan), rabbit anti-human HO-1 antibody (1:500, Enzo Life Sciences, Farmingdale, NY), rabbit anti-human fibrin/fibrinogen antibody (1:4000, Dako, Denmark), and rat anti-mouse MOMA2 antibody (1:400, AbD Serotec, Raleigh, NC) were used. TUNEL stain kit was from Wako chemicals (Osaka, Japan). Neutrophils were visualized using Naphthol AS-D chloroacetate Esterase stain (Muto Pure Chemicals, Tokyo, Japan). About 5 consecutive fields were examined in each slide at 100 or 200-fold magnification. All assessments were performed with ImageJ (National Institutes of Health, Bethesda, MD).

### Statistical Analyses

Multiple groups were compared using two-way ANOVA with the Tukey-Kramer test for parametric values, if necessary logarithm transition was performed. Otherwise, Kruskal-Wallis test with Dunn’s test was used for non-parametric values. All analyses were performed using JMP 11.0.0 (SAS Institute Inc., Cary, NC). Values are presented as mean ± s.e.m or box plot. Differences were considered statistically significant with P < 0.05.

## Electronic supplementary material

Supporting information

## References

[1] Olsson AK, Dimberg A, Kreuger J, Claesson-Welsh L (2006). VEGF receptor signalling - in control of vascular function. Nat Rev Mol Cell Biol.

[2] Walter TJ, Cast AE, Huppert KA, Huppert SS (2014). Epithelial VEGF signaling is required in the mouse liver for proper sinusoid endothelial cell identity and hepatocyte zonation *in vivo*. Am J Physiol Gastrointest Liver Physiol.

[3] Shimizu H (2001). Vascular endothelial growth factor secreted by replicating hepatocytes induces sinusoidal endothelial cell proliferation during regeneration after partial hepatectomy in rats. J Hepatol.

[4] Iacovelli R (2014). Incidence and relative risk of hepatic toxicity in patients treated with anti-angiogenic tyrosine kinase inhibitors for malignancy. Br J Clin Pharmacol.

[5] Hod, T., Cerdeira, A. S. & Karumanchi, S. A. Molecular Mechanisms of Preeclampsia. *Cold Spring Harb Perspect Med* **5**, 10.1101/cshperspect.a023473 (2015). 10.1101/cshperspect.a023473 PMC4588136 26292986

[6] Maynard SE (2003). Excess placental soluble fms-like tyrosine kinase 1 (sFlt1) may contribute to endothelial dysfunction, hypertension, and proteinuria in preeclampsia. J Clin Invest.

[7] Westbrook RH, Dusheiko G, Williamson C (2016). Pregnancy and liver disease. J Hepatol.

[8] Venkatesha S (2006). Soluble endoglin contributes to the pathogenesis of preeclampsia. Nat Med.

[9] Mahasreshti PJ (2003). Intravenous delivery of adenovirus-mediated soluble FLT-1 results in liver toxicity. Clin Cancer Res.

[10] Tam BY (2006). VEGF modulates erythropoiesis through regulation of adult hepatic erythropoietin synthesis. Nat Med.

[11] Gélinas DS, Bernatchez PN, Rollin S, Bazan NG, Sirois MG (2002). Immediate and delayed VEGF-mediated NO synthesis in endothelial cells: role of PI3K, PKC and PLC pathways. Br J Pharmacol.

[12] Leonardo DP (2015). Association of Nitric Oxide Synthase and Matrix Metalloprotease Single Nucleotide Polymorphisms with Preeclampsia and Its Complications. PLoS One.

[13] Zeng F (2016). Associations between nitric oxide synthase 3 gene polymorphisms and preeclampsia risk: a meta-analysis. Sci Rep.

[14] Burke SD (2016). Soluble fms-like tyrosine kinase 1 promotes angiotensin II sensitivity in preeclampsia. J Clin Invest.

[15] Li F (2012). eNOS deficiency acts through endothelin to aggravate sFlt-1-induced pre-eclampsia-like phenotype. J Am Soc Nephrol.

[16] Taniai H (2004). Susceptibility of murine periportal hepatocytes to hypoxia-reoxygenation: role for NO and Kupffer cell-derived oxidants. Hepatology.

[17] Elias-Miró M, Jiménez-Castro MB, Rodés J, Peralta C (2013). Current knowledge on oxidative stress in hepatic ischemia/reperfusion. Free Radic Res.

[18] Gallos ID (2013). Pre-eclampsia is associated with, and preceded by, hypertriglyceridaemia: a meta-analysis. BJOG.

[19] Enquobahrie DA (2004). Maternal plasma lipid concentrations in early pregnancy and risk of preeclampsia. Am J Hypertens.

[20] Dusse LM, Rios DR, Pinheiro MB, Cooper AJ, Lwaleed BA (2011). Pre-eclampsia: relationship between coagulation, fibrinolysis and inflammation. Clin Chim Acta.

[21] Ma L (2005). *In vitro* procoagulant activity induced in endothelial cells by chemotherapy and antiangiogenic drug combinations: modulation by lower-dose chemotherapy. Cancer Res.

[22] Usui J (2014). Clinicopathological spectrum of kidney diseases in cancer patients treated with vascular endothelial growth factor inhibitors: a report of 5 cases and review of literature. Hum Pathol.

[23] Eremina V (2008). VEGF inhibition and renal thrombotic microangiopathy. N Engl J Med.

[24] Kuo KK (2013). NO donor KMUP-1 improves hepatic ischemia-reperfusion and hypoxic cell injury by inhibiting oxidative stress and pro-inflammatory signaling. Int J Immunopathol Pharmacol.

[25] Tateya S (2011). Endothelial NO/cGMP/VASP signaling attenuates Kupffer cell activation and hepatic insulin resistance induced by high-fat feeding. Diabetes.

[26] Kawachi S (2000). Nitric oxide synthase and postischemic liver injury. Biochem Biophys Res Commun.

[27] Wei K (2013). A liver Hif-2α-Irs2 pathway sensitizes hepatic insulin signaling and is modulated by Vegf inhibition. Nat Med.

[28] Rosmorduc O, Housset C (2010). Hypoxia: a link between fibrogenesis, angiogenesis, and carcinogenesis in liver disease. Semin Liver Dis.

[29] Nath B (2011). Hepatocyte-specific hypoxia-inducible factor-1α is a determinant of lipid accumulation and liver injury in alcohol-induced steatosis in mice. Hepatology.

[30] Qu A (2011). Hypoxia-inducible transcription factor 2α promotes steatohepatitis through augmenting lipid accumulation, inflammation, and fibrosis. Hepatology.

[31] Sparkenbaugh EM (2011). The role of hypoxia-inducible factor-1α in acetaminophen hepatotoxicity. J Pharmacol Exp Ther.

[32] Wetzka B (1999). Altered lipid metabolism in preeclampsia and HELLP syndrome: links to enhanced platelet reactivity and fetal growth. Semin Thromb Hemost.

[33] Foley EM (2013). Hepatic remnant lipoprotein clearance by heparan sulfate proteoglycans and low-density lipoprotein receptors depend on dietary conditions in mice. Arterioscler Thromb Vasc Biol.

[34] Williams KJ (2008). Molecular processes that handle–and mishandle–dietary lipids. J Clin Invest.

[35] Erpenbeck L (2016). ADAMTS13 Endopeptidase Protects against Vascular Endothelial Growth Factor Inhibitor-Induced Thrombotic Microangiopathy. J Am Soc Nephrol.

[36] Iafrati MD (2005). Compensatory mechanisms influence hemostasis in setting of eNOS deficiency. Am J Physiol Heart Circ Physiol.

[37] Tojo Y (2015). Hypoxia Signaling Cascade for Erythropoietin Production in Hepatocytes. Mol Cell Biol.

[38] Parnas M (2006). Moderate to severe thrombocytopenia during pregnancy. Eur J Obstet Gynecol Reprod Biol.

[39] Kumasawa K (2011). Pravastatin induces placental growth factor (PGF) and ameliorates preeclampsia in a mouse model. Proc Natl Acad Sci USA.

[40] Van Steenkiste C (2011). Inhibition of placental growth factor activity reduces the severity of fibrosis, inflammation, and portal hypertension in cirrhotic mice. Hepatology.

[41] Li, X. *et al*. Placental growth factor silencing ameliorates liver fibrosis and angiogenesis and inhibits activation of hepatic stellate cells in a murine model of chronic liver disease. *J Cell Mol Med*, 10.1111/jcmm.13158 (2017). 10.1111/jcmm.13158 PMC5618674 28378526

[42] Autiero M, Luttun A, Tjwa M, Carmeliet P (2003). Placental growth factor and its receptor, vascular endothelial growth factor receptor-1: novel targets for stimulation of ischemic tissue revascularization and inhibition of angiogenic and inflammatory disorders. J Thromb Haemost.

[43] Zhou CC (2008). Angiotensin receptor agonistic autoantibodies induce pre-eclampsia in pregnant mice. Nat Med.

[44] Kanasaki K (2008). Deficiency in catechol-O-methyltransferase and 2-methoxyoestradiol is associated with pre-eclampsia. Nature.

[45] Li F (2016). Nicotinamide benefits both mothers and pups in two contrasting mouse models of preeclampsia. Proc Natl Acad Sci USA.

[46] Takahashi N, Boysen G, Li F, Li Y, Swenberg JA (2007). Tandem mass spectrometry measurements of creatinine in mouse plasma and urine for determining glomerular filtration rate. Kidney Int.

[47] Oe Y (2016). Coagulation Factor Xa and Protease-Activated Receptor 2 as Novel Therapeutic Targets for Diabetic Nephropathy. Arterioscler Thromb Vasc Biol.

[48] Hayashi S (2017). Protease-activated receptor 2 exacerbates adenine-induced renal tubulointerstitial injury in mice. Biochem Biophys Res Commun.

[49] Brunt EM, Tiniakos DG (2010). Histopathology of nonalcoholic fatty liver disease. World J Gastroenterol.

