# Supplementary material for: Hepatic dysfunction and thrombocytopenia induced by excess sFlt1 in mice lacking endothelial nitric oxide synthase
Source: Sci Rep. 2018 Jan 8;8:102. doi: 10.1038/s41598-017-18260-7 (PMC5758763; doi:10.1038/s41598-017-18260-7)
Supplement: Supplementary file 1 — Supporting information [file 41598_2017_18260_MOESM1_ESM.pdf]

# Supplemental Material

# Hepatic dysfunction and thrombocytopenia induced by excess sFlt1 in mice lacking endothelial nitric oxide synthase

Yuji Oe[1,5], Mieko Ko[2], Tomofumi Fushima[2], Emiko Sato[2,3], S Ananth Karumanchi[4], Hiroshi Sato[2,3], Junichi Sugawara[1], Sadayoshi Ito[3], and Nobuyuki Takahashi[2,3]*

[1] Division of Feto-Maternal Medical Science, Department of Community Medical Support, Tohoku Medical Megabank Organization, Tohoku University, Sendai 980-8574, Japan.

[2] Division of Clinical Pharmacology and Therapeutics, Tohoku University Graduate School of Pharmaceutical Sciences & Faculty of Pharmaceutical Sciences, Sendai 980-8578, Japan.

[3] Division of Nephrology, Endocrinology, and Vascular Medicine, Tohoku University Graduate School of Medicine, Sendai 980-8574, Japan.

[4] Department of Medicine and Center for Vascular Biology Research, Beth Israel Deaconess Medical Center and Harvard Medical School, Boston, MA 02215, USA.

[5] Research Fellow of Japan Society for the Promotion of Science.

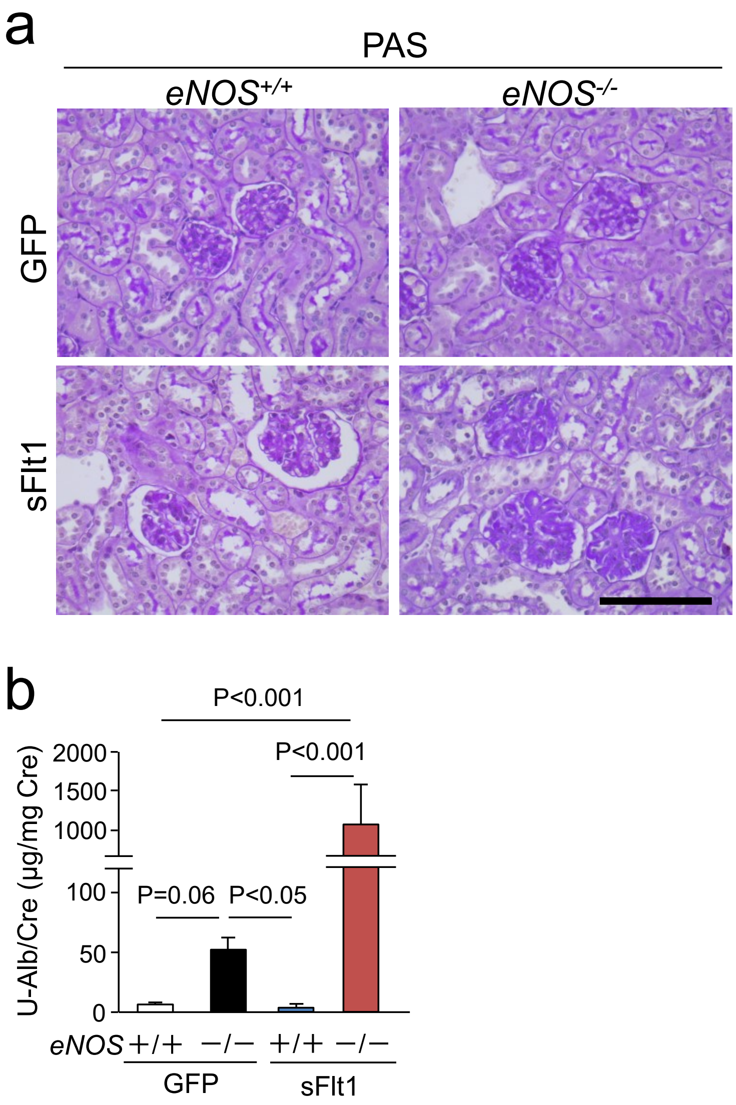


**Supplementary Figure 1.** Glomerular injury and urinary albumin excretion in *eNOS*$^{-/-}$; sFlt1 mice. (**a**) Representative photomicrographs of Periodic acid-Schiff (PAS) stain in the kidney. Severe endotheliosis accompanied by mesangial expansion is shown in glomeruli from *eNOS*$^{-/-}$; sFlt1 mice. Scale bar indicates 100 µm. (**b**) The level of urinary albumin/creatinine ratio (U-Alb/Cre) in each group. n=6-8. Data are shown as mean ± s.e.m. ANOVA.

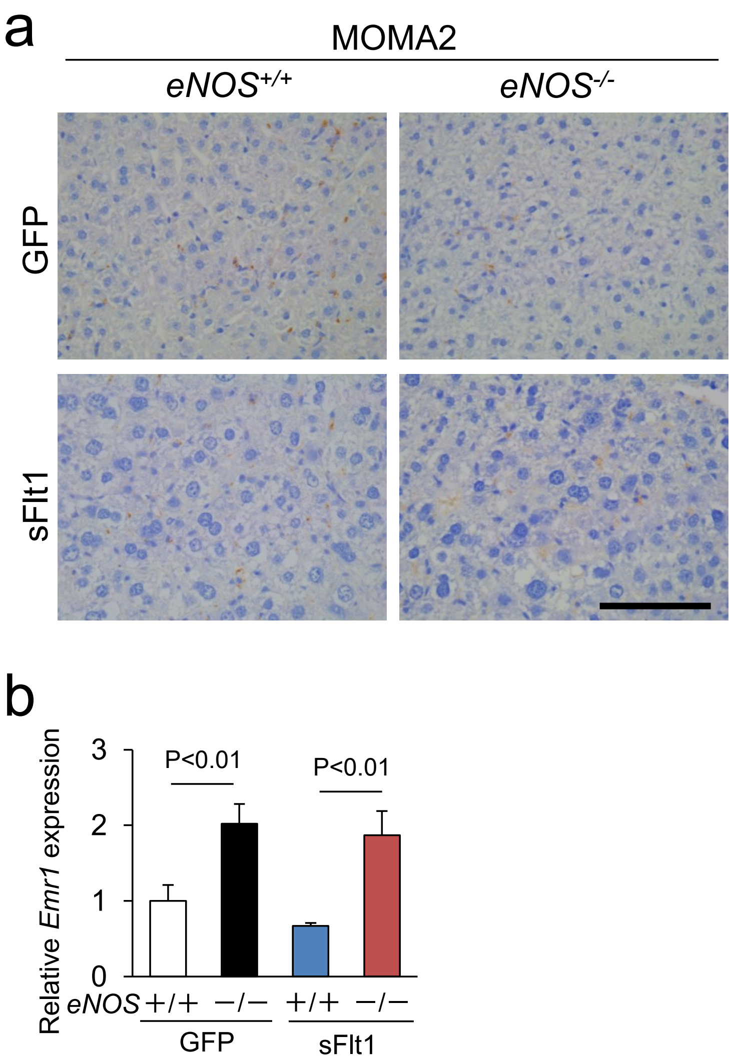


**Supplementary Figure 2.** Excessive sFlt1 do not affect macrophage infiltration.
(**a**) Representative photomicrographs of MOMA-2, a macrophage marker, in the liver. Scale bar indicates 100 µm. (**b**) Expression of *Emr1* mRNA in the liver. n=7-8. Data are shown as mean ± s.e.m. ANOVA.

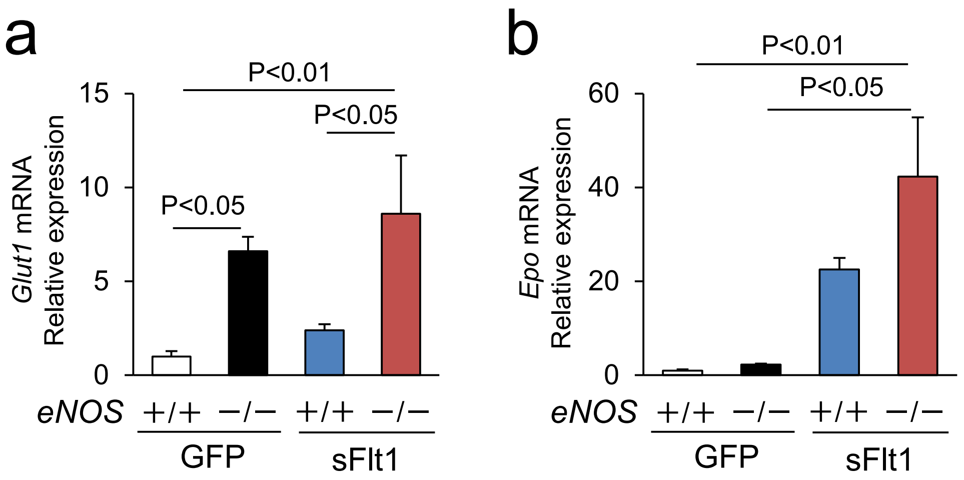


**Supplementary Figure 3.** Expression of *Glut1* and *Epo* mRNA in the liver
(**a-b**) the levels of *Glut1* and *Epo* mRNA in the liver. n=7-8. Data are shown as mean ± s.e.m. ANOVA.

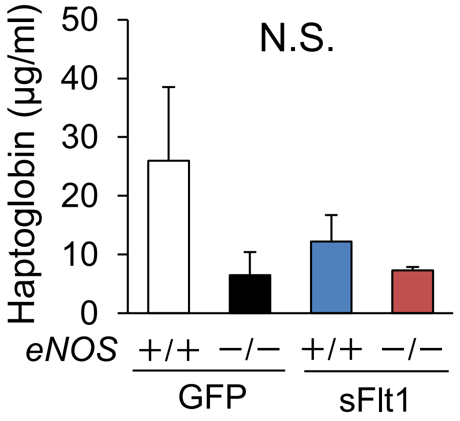


**Supplementary Figure 4.** The level of plasma haptoglobin in each group. n=7-8. Data are shown as mean ± s.e.m.

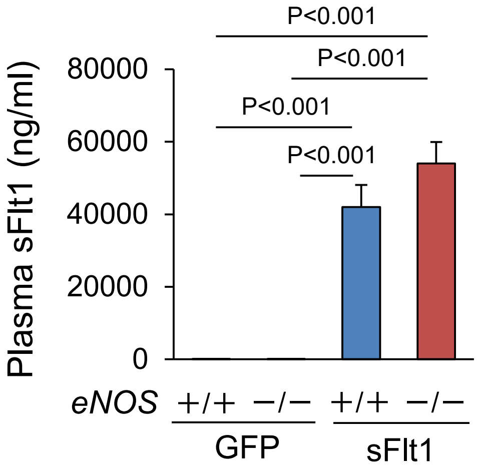


**Supplementary Figure 5.** The level of plasma sFlt1 in each group. n=7-8. Data are shown as mean ± s.e.m. ANOVA
